# Rope skipping increases bone mineral density at calcanei of pubertal girls in Hong Kong: A quasi-experimental investigation

**DOI:** 10.1371/journal.pone.0189085

**Published:** 2017-12-08

**Authors:** Amy S. Ha, Johan Y. Y. Ng

**Affiliations:** Department of Sports Science and Physical Education, The Chinese University of Hong Kong, Shatin, Hong Kong; CUNY, UNITED STATES

## Abstract

Bone mineral accrual during puberty is important, especially in girls, because it is related to reduced risks of osteoporosis in adulthood. Previous research has shown that jumping or plyometric exercises may be effective in increasing bone mineral density in adolescents. Rope skipping is a form of activity that involves jumping, thus regular skipping may also increase bone mineral density in pubertal girls. To this end, we conducted a quasi-experimental to examine the effects of rope skipping on girls’ bone mineral density and cardiovascular fitness. 176 Hong Kong girls (age = 12.23 ± 1.80 years at baseline) were recruited to take part in the study. Bone density at their forearms and calcanei were measured twice over two academic years (mean time between visits was 10.3 months). Using multilevel modeling analyses and adjusting for participants’ height and physical activity, we found that girls who participated in weekly rope skipping activities, compared to those who did not, had higher levels of bone density at the calcanei (*B* = 0.023, *p* < .01). However, no differences were found for bone density at forearms or participants’ cardiovascular fitness. The rates of change of these variables across time were also not significantly different. Results suggest that regular rope skipping may increase girls’ bone density at the lower extremities, irrespective of the amount of self-report physical activity. However, further research is required to examine the potential dose-response relation between skipping behaviors and the measured outcomes.

## Introduction

Many older adults, especially women, are affected by osteoporosis or related injuries, such as bone fracture from falls [1]. Although the problem often occurs during individuals’ later stages of life, preventive measures could be implemented during childhood and adolescence, as it was found that girls have the greatest bone mineral accrual rates during these periods [2–4]. Maximizing the rate of bone mineral accrual during adolescence may therefore increase the peak bone mineral density (BMD) of girls [5], which is a strong predictor of the same variable at adulthood [6]. Interventions based on weight-bearing exercises, and designed to enhance BMD, were found to be effective for girls [7–10]. Specifically, exercise-based interventions designed to improve BMD in girls have often involved jumping [11–13] or plyometric exercises [14]. In particular, rope skipping is also a form of plyometric exercise, which may be an appropriate form of exercise that could be used to improve BMD.

Apart from girls’ bone health, their physical fitness also plays a role in their current and future health [15,16]. Specifically, these researchers found that cardiovascular fitness of children and adolescents is related to healthier body composition, lower cardiovascular disease risk factors, and quality of life. In particular, as rope skipping is a relatively vigorous form of physical activity [17], it may also contribute to better cardiovascular fitness in rope skippers.

Based on the above, we propose that intermediate to advanced rope skipping is an activity that could potentially improve the bone health and cardiovascular fitness of pubertal girls. Moreover, some intermediate to advanced levels rope skipping tricks involve high impact movements to the upper limbs (e.g., push-ups, cartwheels). Therefore, regular rope skipping may enhance bone mineral accrual at both the upper and lower limbs of girls. To our knowledge, the specific effects of rope skipping on these outcomes have not been explored previously. Therefore, we designed a quasi-experimental study to examine the benefits of rope skipping in terms of girls’ bone mineral density and cardiovascular fitness. Specifically, we compared bone mineral density and cardiovascular fitness of a group of girls who participated in rope skipping regularly, versus those who did not. Outcomes of participants were measured twice within a 12-month period, so that we could also compare the rates of changes in these outcomes over this period. Results were adjusted for other factors that were previously found to be related to these outcomes, including age, height, pubertal stages, and physical activity levels [3,18]. We hypothesized girls in the experimental group (i.e., those who participate in rope skipping regularly), compared to those in the control group, will show significantly higher levels of BMD (measured at the forearms and calcanei) and cardiovascular fitness scores at follow-up, after adjusting for potential confounding outcomes. We also hypothesized that girls in the experimental group will show higher rates of change in these outcomes from baseline to follow-up.

## Method

### Participants and procedures

A total of 176 Hong Kong girls (12.23 years, *SD* = 1.80 at baseline) were recruited to take part in the study. Written informed consent for participation was sought from all participants and their parents prior to the study. Participants were recruited from local schools in Hong Kong; all participants were Chinese. The experimental group (*n* = 66; age = 12.45 years, *SD* = 1.67 at baseline) included girls who were members of school rope skipping or regular rope skipping classes, who took part in rope skipping-related activities for at least one hour weekly. Other participants (*n* = 110; age = 12.09 years, *SD* = 1.86 at baseline) were included in the control group. Participants were invited to have their measurements taken during baseline and follow-up, respectively, at the authors’ institution. These included the completion of a questionnaire, and measurements for BMD and cardiovascular fitness. Participants were invited to attend two measurement sessions over two academic years. Out of all participants who provided baseline measures, 143 also attended during follow-up, representation an overall dropout rate of 19%. All baseline measures for girls who dropped out were compared to those who attended measurement sessions at both time points using one-way ANOVAs. No differences in any of the variables were found. The mean time between baseline and follow-up visits was 314.7 days (approximately 10.3 months). Participants were asked to self-report whether they engaged in regular (i.e., at least one 1-hour session per week) rope skipping during the past three months. All participants in the experimental group reported regular rope skipping at both baseline and follow-up. Similarly, all participants in the control group reported no regular rope skipping at both time points. All participants received a HK$50 transportation subsidy for each completed visit at baseline and follow-up. All study procedures were reviewed and approved by the Joint Chinese University of Hong Kong-New Territories East Cluster Clinical Research Ethics Committee (Ref. no.: 2014.216).

### Measures

#### Height and weight

Participants’ standing height was measured by research assistants using a portable standiometer. Their weights were measured using an electronic scale with precision up to 0.1 kg.

#### Pubertal stage

Participants’ pubertal (Tanner) stages were measured using self-reported scale by Chan and colleagues [19]. Based on their responses for this 6-item measure, participants will be characterized as prepubertal (Tanner stage I), early pubertal (Tanner stage II), midpubertal (Tanner stage III), late pubertal (Tanner stage IV), or postpubertal (Tanner stage V). As the relation between pubertal stages and the measured outcomes are not expected to be linear, participants’ Tanner stage was used as a categorical outcome in all analyses.

#### Physical activity

The Chinese version of the Physical Activity Questionnaire-Children [20,21] was administered to participants as a measure for their physical activity during school recesses, lunch periods, and during their leisure time. Questionnaire responses were used to calculate a physical activity score for each respondent. This score has a possible range from 1 (doing no physical activity at all) to 5 (being active every day), with larger values indicating higher physical activity levels. The physical activity score was used as a control variable in the main analyses.

#### Bone mineral density

Participants’ bone mineral density was measured using dual-energy X-ray absorptiometry (DEXA). Measurements were taken at two sites, namely the forearms and calcanei, using the OsteoSys EXA-3000 (OsteoSys Corp., Seoul, Korea) device. In this study, the mean of measures taken at the right and left forearms for the same participant was used as a combined forearm score (i.e., BMD-forearm). Similarly, a combined BMD-calcanei score was calculated as the mean of BMD values taken at the left and right calcaneus, respectively. All bone mineral density data were measured by a trained research assistant, under the supervision of the authors of this paper.

#### Cardiovascular fitness

A 15-meter multistage shuttle run test was used to assess participants’ cardiovascular fitness [22]. Specifically, participants were asked to complete laps of a shuttle run following a pre-recorded cadence. The test will stop upon exhaustion or the participant failing to follow the cadence twice. The number of successfully completed laps was used as a measure for participants’ cardiovascular fitness.

### Data analyses

Two-level (time within participant), random-slope random-intercept, multiple regressions were used to examine the study hypotheses. Analyses were conducted using MLwiN version 2.26. Specifically, the variables of Time (0 = baseline, 1 = follow-up) and Group (0 = control, 1 = experimental) were included in the equations to test whether participants outcomes changed from baseline to follow, and if there were differences between experimental and control groups, respectively. Time × Group interaction terms were also added to examine whether the rates of change in the variables differed between groups.

Based on previous studies [14,18,23–25], the BMD outcomes and cardiovascular fitness were adjusted for associated factors including girls’ height and physical activity. The interaction term Time × Tanner Stage was also included to account for variations in growth rates at different pubertal stages. Given the limited sample size per Tanner stage in the analyses, the inclusion of the interaction term should merely be used as statistical adjustment, and should not be used for drawing any conclusions.

## Results

### Preliminary analyses

Descriptive statistics of participants’ anthropometric measurements and outcome measures are shown in Table 1. Using one-way ANOVAs, we found that girls’ in the experimental group, compared to those in the control group, reported higher levels of physical activity at both time points (*p*s < .01). However, only those in the experimental group showed a decrease in physical activity from baseline to follow-up (*p* = .03). In terms of BMD, we found that the BMD-forearm of girls from both experimental (*p* = .04) and control (*p* = .02) groups increased from baseline to follow-up. Between group differences were not found at baseline (*p* = .06). At follow-up, the experimental group had higher BMD-forearm than the control group (*p* = .05). For BMD-calcanei, neither groups showed increases from baseline to follow-up (*p*s = .09 and .12 for experimental and control group, respectively). However, at both time points, the experimental group had higher levels of BMD-calcanei than the control group (*p*s < .01). For cardiovascular fitness scores, no between-group differences or changes from baseline to follow-up were found.

**Table 1:** Descriptive statistics of measured outcomes.

	Baseline			Follow-up		
	Experimental (*n* = 66)	Control (*n* = 110)	All (*n* = 176)	Experimental (*n* = 46)	Control (*n* = 97)	All (*n* = 143)
Age	12.45 ± 1.67^c^	12.09 ± 1.86^c^	12.23 ± 1.80^c^	13.29 ± 1.76^c^	12.92 ± 1.85^c^	13.04 ± 1.82^c^
Height (cm)	150.97 ± 9.82^c^	149.08 ± 10.10^c^	149.77 ± 10.01^c^	154.82 ± 6.73^c^	153.24 ± 8.87^c^	153.74 ± 8.25^c^
Weight (kg)	43.60 ± 9.59^c^	42.06 ± 11.38^c^	42.63 ± 10.75^c^	47.24 ± 8.12^c^	45.49 ± 11.74^c^	46.05 ± 10.71^c^
Tanner stages						
Prepubertal (Stage I)	9	11	20	1	5	6
Early pubertal (Stage II)	8	16	24	4	14	18
Midpubertal (Stage III)	12	28	40	7	14	21
Late pubertal (Stage IV)	33	42	75	31	55	86
Postpubertal (Stage V)	2	3	5	2	6	8
Missing	2	10	12	1	3	4
Physical activity	2.78 ± 0.55^a^^,^^c^	2.29 ± 0.57^a^	2.48 ± 0.61^c^	2.52 ± 0.65^b^^,^^c^	2.21 ± 0.61^b^	2.31 ± 0.64^c^
BMD–forearm (g cm^-1^)	0.222 ± 0.094^c^	0.193 ± 0.096^c^	0.207 ± 0.096^c^	0.264 ± 0.102^b^^,^^c^	0.227 ± 0.100^b^^,^^c^	0.238 ± 0.102^c^
BMD–calcanei (g cm^-1^)	0.447 ± 0.084^a^	0.406 ± 0.093^a^	0.421 ± 0.092	0.473 ± 0.081^b^	0.427 ± 0.077^b^	0.441 ± 0.081
Cardiovascular fitness (stages)	31.56 ± 16.64	28.81 ± 16.02	29.83 ± 16.26	28.89 ± 15.56	27.78 ± 12.82	28.14 ± 13.73

^a^Between-group difference at baseline (*p* < .05).

^b^Between-group difference at follow-up (*p* < .05).

^c^Significant difference between scores at baseline and follow-up (*p* < .05).

Pearson correlations between measured variables are presented in Table 2. In particular, and in line with results from previous studies, we found participants’ height to be associated with their BMD at both measured sites, and at both time points (*r*s = .49 to .70, *p*s < .01). Physical activity was related to BMD-calcanei at baseline (*r* = .18, *p* = .02). However, neither the corresponding correlation at follow-up (*p* = .08), nor the associations with BMD-forearm at either time points (*p* > .91) were significant. Using one-way ANOVAs, we also found that, at both time points, there were differences in both BMD outcomes between participants’ pubertal stage (*p*s < .01). For cardiovascular outcomes, as expected, we found physical activity to be a potential confounding factor (*r*s = .38 and .25, *p*s < .01, for baseline and follow-up, respectively). These preliminary results support our decision to use the selected outcomes as adjusting variables in our main analyses.

**Table 2:** Pearson correlation between measured outcomes.

		1		2		3		4		5		6		7	
1.	Age			.82	**	.63	**	-.12		.75	**	.55	**	.19	*
2.	Height	.72	**			.77	**	-.05		.70	**	.61	**	.24	**
3.	Weight	.54	**	.71	**			-.01		.65	**	.69	**	.06	
4.	Physical activity	-.21	*	-.06		.03				.01		.18	*	.38	**
5.	BMD–forearm	.63	**	.57	**	.52	**	.01				.67	**	.24	**
6.	BMD–calcanei	.48	**	.49	**	.66	**	.15		.62	**			.19	*
7.	Cardiovascular fitness	.20	*	.13		-.03		.25	**	.21	*	.12			

* *p* < .05

** *p* < .01.

Correlations at baseline are placed above the diagonal; those at follow-up are placed below the diagonal.

#### Main analyses

Using a two-level regression analysis, we found that girls’ BMD-forearm did not increase from baseline to follow-up (*B* = 0.005, *p* = .70, 95% CI [-0.020, 0.029]). The Group (*B* = 0.012, *p* = .26, 95% CI [-0.009, 0.032]) or the Time × Group (*B* = 0.003, *p* = .78, 95% CI [-0.018, 0.024]) terms were also not significant, suggesting the groups did not differ in follow-up scores and in terms of the changes in the outcome, respectively. As hypothesized, participants’ height was related to their BMD-forearm measures (*B* = 0.006, *p* < .01, 95% CI [0.004, 0.007]). Detailed results from the main analyses are presented in Table 3.

**Table 3:** Results of two-level regression analyses.

Regression terms	BMD-forearm			BMD-calcanei			Cardiovascular fitness		
	*B*	*SE*	*p*	*B*	*SE*	*p*	*B*	*SE*	*p*
Intercept	-0.631	0.100	< .01	-0.414	0.094	< .01	-40.19	18.81	.03
Time	0.005	0.012	.70	0.004	0.011	.69	-3.33	2.94	.26
Group	0.012	0.010	.26	0.023	0.008	< .01	-0.52	1.84	.78
Time × Group	0.003	0.011	.78	0.006	0.008	.47	1.48	2.24	.51
Height	0.006	0.001	< .01	0.005	0.001	< .01	0.37	0.12	< .01
Physical activity	0.003	0.007	.70	0.012	0.006	.04	4.73	1.31	< .01
Time × Tanner Stage I	Reference term			Reference term			Reference term		
Time × Tanner Stage II	0.019	0.014	.16	-0.006	0.013	.63	-1.06	3.55	.76
Time × Tanner Stage III	0.019	0.012	.12	-0.002	0.011	.84	-2.01	3.23	.53
Time × Tanner Stage IV	0.005	0.013	.69	0.004	0.011	.72	-0.64	3.11	.84
Time × Tanner Stage V	0.005	0.023	.84	-0.016	0.021	.44	10.01	5.60	.07

In terms of BMD at the calcanei, the regression terms for Time (*B* = 0.004, *p* = .69, 95% CI [-0.017, 0.025]) and Time × Group (*B* = 0.006, *p* = .47, 95% CI [-0.010, 0.023]) were also not significant. However, in support with our hypothesis, we found that girls in the experimental group, compared to those in the control group, had higher levels of BMD-calcanei at follow-up (*B* = 0.023, *p* < .01, 95% CI [0.007, 0.038]). The terms for the adjusting variables of Height (*B* = 0.005, *p* < .01, 95% CI [0.004, 0.007]) and Physical activity (*B* = 0.012, *p* = .04, 95% CI [0.001, 0.023]) were also significant.

Regarding cardiovascular fitness, the terms in the regression model for Time (*B* = -3.33, *p* = .26, 95% CI [-9.10, 2.44]), Group (*B* = -0.52, *p* = .78, 95% CI [-4.14, 3.10]), and Time × Group (*B* = 1.48, *p* = .51, 95% CI [-2.92, 5.89]) were not significant. Nonetheless, we found that participants’ Height and Physical activity was related to their performances in the cardiovascular fitness test (*B* = 0.37, *p* < .01, 95% CI [0.14, 0.61] and *B* = 4.73, *p* < .01, 95% CI [2.15, 7.31] for Height and Physical activity, respectively).

## Discussion

We conducted the study to examine the effects of rope skipping on girls’ physical outcomes, including their BMD and cardiovascular fitness. For the three main outcomes of BMD measured at the forearms and calcanei, and also participants’ cardiovascular fitness, we found that there were no between-group differences in terms of the rates of change in these variables from baseline to follow-up. In support for our hypotheses, we found that girls who took part in rope skipping activities regularly, compared to those who did not, had higher levels of BMD at the calcanei. This result support the notion that rope skipping may be an activity that is beneficial to students’ bone mineral levels at their lower extremities. This is unsurprising as previous studies have also showed that jumping or plyometric exercises may increase participants’ BMD [8,26]. Nonetheless, participants in the experimental group also had higher levels of calcanei BMD at baseline. This may be attributed to the fact that girls in the experimental have already been skipping regularly before baseline measures were taken. Researchers have previously suggested that bone mineral accrual could be accelerated only if the level of loading exceeded the accustomed levels [27]. It is possible that girls in the experimental group have higher levels of calcanei BMD due to the additional loading from the rope skipping activities, but at follow-up, their bodies might have adapted to the level of loading, and hence we found no differences in terms of the rates of change in BMD between the two groups.

Dissimilar to previous results, the same effect of rope skipping was not found for BMD measured at the forearm. Relative to some other activities, rope skipping may include less impact or weight-bearing movement on the upper extremities, this might be a possible explanation for the null findings. Moreover, there were no significant increases from baseline to follow-up in bone density measured at both sites, after adjusting for covariates in our main analyses. This finding was unexpected given that researchers have shown that there were increases in BMD during girls’ puberty [3,5,6]. In fact, we did find in our preliminary analyses that overall levels of BMD in participants’ forearms appeared to be higher in follow-up than at baseline. One plausible explanation for these results is that girls’ height, which increased from baseline to follow-up as expected, was strongly related to their BMD in terms of Pearson correlations, and therefore the effects of Time may have been reduced due to multicollinearity issues. Another potential explanation is that the time between baseline and follow-up visits may differ for each participant. This might have introduced additional variations to the results.

In terms of cardiovascular fitness, our hypotheses were also not supported by our results. Specifically, participants’ fitness was not different across groups; their rate of change in this outcome was also not significant. This suggests that rope skipping did not contribution to girls’ cardiovascular fitness, as hypothesized. Nonetheless, we found from our main analyses that physical activity was a strong predictor of cardiovascular fitness. Therefore, in line with previous results, there seems to be a dose-response relation between physical activity and fitness [28], but perhaps less so for the type of activity being done. It is possible that there could be also be a dose-response relation between the frequency and intensity of rope skipping activities with fitness outcomes. Unfortunately, these parameters regarding rope skipping behaviors were not measured in this study.

One limitation of our study concerns the research design used. Specifically, as in all quasi-experiments, non-randomized group allocation increases the likelihood of finding differences in key outcomes at baseline. In this study, between-group differences at baseline was observed for participants’ physical activity and BMD at calcanei. Consequently, we adopted a multilevel approach for data analyses, which provides adjustments for baseline differences. Nonetheless, to provide further support for rope skipping as an effective way to increase girls’ BMD, experimental designs with randomized group allocation, with all participants having no prior intervention, should be used in future studies. Researchers may also apply more rigorous protocols for the rope skipping programs utilized in future studies.

The majority of published studies used whole-body DEXA to measures participants’ BMD [9,14]. Instead, in our study, we used a more localized tool for such measures. The differences in these methods would reduce the comparability of the results. However, localized measurement tools, such as the one used in our study or similar ultrasound-based equipment [13], might have increased ease of measurement and may be more time-efficient. Consequently, these measures might be appropriate for studies involving a large number of participants. Another limitation of the study is that we did not measure, and in turn adjust for some potential confounding variables included in previous studies, such as lean mass and fat mass [26]. In particular, researchers have found that lean mass is a strong predictor of BMD [29], which was not measured in our study. Nevertheless, we have made adjustments for outcomes that we considered to be important and relevant. Further, adjusting for too many factors may also lead to inflated statistical errors [30]. Therefore, including only a modest set of covariates in our analyses should not be problematic. Finally, information regarding participants’ frequency and intensity of rope skipping and other physical activity should be measured in similar future studies. This will allow researchers to examine whether there is a dose-response relation between these parameters and BMD or cardiovascular fitness. The use of objective measures, such as accelerometry for physical activity, could also reduce the potential biases from self-reporting, and therefore should be considered.

In conclusion, results from this study suggest that girls’ bone health and cardiovascular fitness is related to their physical growth and the amount of physical activity they take part in. In addition, our results suggest that rope skipping contributes to bone health at the lower limbs, and could be included as part of general physical activity. Nonetheless, evidence pertaining the dose-response relation between the frequency, duration, and intensity of rope skipping, and BMD outcomes is still required.
